# Supplementary material for: Wound Restorative Power of Halimeda macroloba/ Mesenchymal Stem Cells in Immunocompromised Rats via Downregulating Inflammatory/Immune Cross Talk
Source: Mar Drugs. 2023 May 30;21(6):336. doi: 10.3390/md21060336 (PMC10301341; doi:10.3390/md21060336)
Supplement: Supplementary file 1 [file marinedrugs-21-00336-s001.zip › marinedrugs-2333700-supplementary.pdf]

Skin healing combined potential of Cytoseira/mesenchymal stem cells in immunosuppressed rats *via* overwhelming immuno-inflammatory cross talk

Table S1: Dereplicated compounds from *Halimeda macroloba* algae

No.	Compound name	Molecular Formula	RT (min)	Ionization (ESI+/ESI-)	Molecular Weight	Observed (*m/z*)	References
**1**	Vanillic acid 4-sulfate	$C_8H_8O_7S$	0.5456	$[M-H]^-$	247.9991	246.9923	[1]
**2**	Catechin (isomer)	$C_{15}H_{14}O_6$	0.6605	$[M-H]^-$	290.079	289.069	[1]
**3**	Dihydro-caffeic acid-4-O-glucuronide	$C_{15}H_{18}O_{10}$	0.7453	$[M-H]^-$	358.0900	357.0908	[2]
**4**	Protocatechuic acid 4-O-glucoside	$C_{13}H_{16}O_9$	0.7491	$[M-H]^-$	316.0794	315.0734	[1]
**5**	3-O-(6'-Sulfo-a-D-quinovopyranosyl)-glycerol	$C_9H_{18}O_{10}S$	0.8517	$[M-H]^-$	318.0607	317.0535	[3]
**6**	4-O-(40-(dimethylamino)-40-iodobutan-50-yl-	$C_{11}H_{25}IN_2O_5$	1.6685	$[M-H]^-$	392.0809	391.0794	[4]

	10,20,30-triol)-N methylbutanamide						
**7**	Cycloshermilamine D	$C_{21}H_{16}N_4OS$	1.8473	$[M - H]^-$	372.1035	371.0963	[5]
**8**	Galloyl glucose	$C_{13}H_{16}O_{10}$	2.1543	$[M - H]^-$	332.0737	331.0694	[2]
**9**	Caffeic acid	$C_9H_8O_4$	2.3264	$[M - H]^-$	180.0422	179.0445	[6]
**10**	Gallocatechin	$C_{15}H_{14}O_7$	4.0892	$[M - H]^-$	306.073	305.0657	[1]
**11**	Helmidatetraacetate	$C_{28}H_{38}O_9$	6.4323	$[M - H]^-$	518.2515	519.2601	[7]
**12**	Halimedatrial	$C_{20}H_{26}O_3$	8.6453	$[M - H]^-$	314.1881	313.1871	[7]
**13**	2-methoxy-6Z-octadecenoic acid	$C_{19}H_{36}O_3$	9.1550	$[M + H]^+$	312.267	313.2742	[8]
**14**	Chlorophyll B	$C_{55}H_{70}MgN_4O_6$	9.8791	$[M - H]^-$	905.4984	906.5056	[9]
**15**	Clionasterol	$C_{29}H_{50}O$	12.4295	$[M + H]^+$	414.3861	415.3981	[10]

**16**	Cholesta-5,22-dien-3beta-ol; 22-Dehydrocholesterol	$C_{27}H_{44}O$	12.4895	$[M + H]^+$	384.3392	385.3465	[11]
**17**	di-(2-Ethylhexyl) phthalate	$C_{24}H_{38}O_4$	13.5920	$[M + H]^+$	390.2771	391.2844	[4]
**18**	Cholestane-3beta,5a-diol-6-one	$C_{27}H_{46}O_3$	14.9125	$[M + H]^+$	418.3435	419.3508	[12]
**19**	24-isopropyl cholesterol	$C_{30}H_{52}O$	17.6926	$[M + H]^+$	428.4018	429.4031	[4]

RT: Retention time; min: Minute

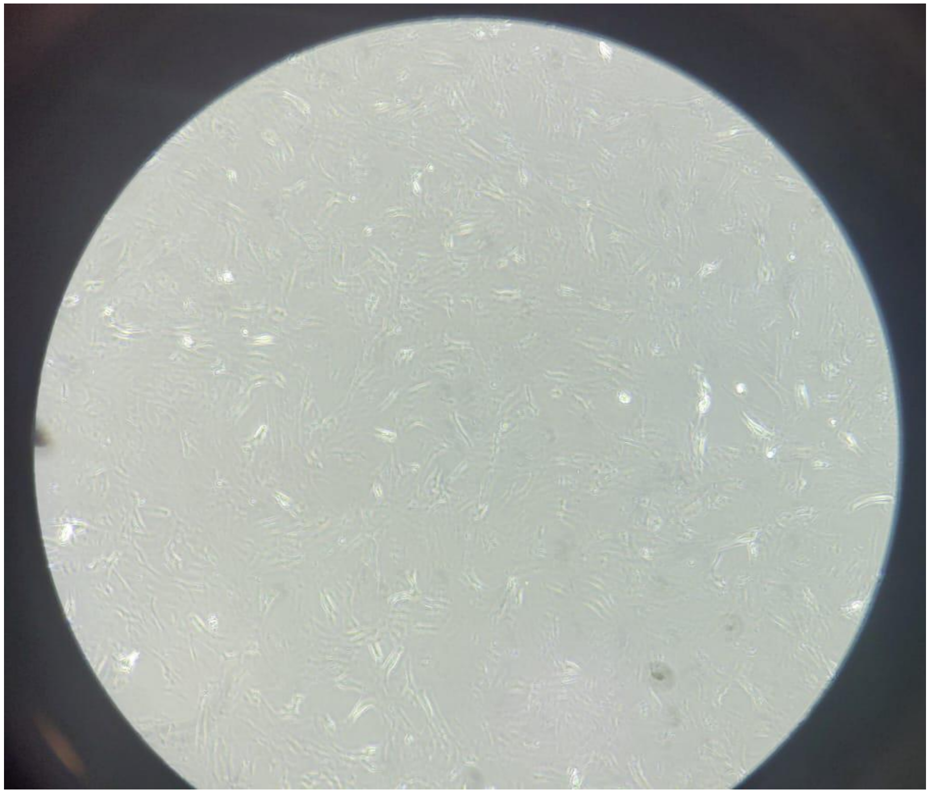

Figure S1. The characterized bone marrow stem cells

Table S2. Primers used for Real time PCR.

Name of gene	Accession number		Primer sequence
IL- 1β	*NC_013670.1*	forward	5'-AGC TTC TCC AGA GCC ACA AC-3'
		reverse	5'-CCT GAC TAC CCT CAC GCA CC-3'
GAPDH	*NC_013676.1*	forward	5'-GTC AAG GCT GAG AAC GGG AA-3'

		reverse	5'-ACA AGA GAG TTG GCT GGG TG-3'
TGF-β	*NC_013672.1*	forward	5'-GAC TGT GCG TTT TGG GTT CC-3'
		reverse	5'-CCT GGG CTC CTC CTA GAG TT-3'
TNF-α	*NC_013680.1*	forward	5'-GAG AAC CCC ACG GCT AGA TG-3'
		reverse	5'-TTC TCC AAC TGG AAG ACG CC-3'
IL-10		forward	5'-TGC CAA GCC TTG TCA GAA ATG ATC AAG-3'
		reverse	5'-GTA TCC AGA GGG TCT TCA GCT TCT CTC-3'
NF-κB		forward	5'-TTA CGG GAG ATG TGA AGA TG-3'
		reverse	5'-ATG ATG GCT AAG TGT AGG AC-3'
INF-ϒ		forward	5'-ATG AGT GCT ACA CGC CGC GTC TTG G-3'
		reverse	5'-GAG TTC ATT GAC AGC TTT GTG CTG G-3'
Cox-1		forward	5'-CCC ACC TTC CGT AGA ACA GG-3'
		reverse	5'-TCC TCC ACA AAC CCA ACG AG-3'
Cox-2		forward	5'-CAT TGA CCA GAG CAG AGA GAT-3'
		reverse	5'-TTC TTG AAT GTC CTC TCT TTC-3'
